# Green Synthesis of Titanium Oxide Nanoparticles With Rosemary and Ginger and Their Bactericidal Action Against Staphylococcus aureus

**DOI:** 10.7759/cureus.45892

**Published:** 2023-09-25

**Authors:** Thamarai Selvi V T, Jerry Joe Chokkattu, Neeharika S, Mahesh Ramakrishnan, Rajeshkumar Shanmugam

**Affiliations:** 1 Prosthodontics, Saveetha Dental College and Hospitals, Saveetha Institute of Medical and Technical Sciences, Saveetha University, Chennai, IND; 2 Pedodontics and Preventive Dentistry, Saveetha Dental College and Hospitals, Saveetha Institute of Medical and Technical Sciences, Saveetha University, Chennai, IND; 3 Pharmacology, Saveetha Dental College and Hospitals, Saveetha Institute of Medical and Technical Sciences, Saveetha University, Chennai, IND

**Keywords:** green synthesis, titanium oxide nanoparticles, ginger, rosemary, staphylococcus aureus

## Abstract

Introduction: Titanium oxide (TiO_2_) nanoparticles (NPs) have significantly proved to be highly useful in restorative materials, dental adhesives, sealants, cements, and other dental applications to prevent microbial colonization and reduce the risk of infections. The present study was aimed at developing a dental material with antibacterial properties by combining titanium oxide NPs using ginger and rosemary extracts.

Materials and methods: The formulation was prepared using rosemary and ginger, mediated by TiO_2_ NPs. The preparation was then introduced into the wells of a microplate consisting of cultured Staphylococcus aureus and was kept for incubation for four hours. To record the minimum inhibitory concentration, the test solution was added into Kimble tubes consisting of Muller-Hinton broth. The results obtained were statistically analyzed using one-way ANOVA.

Result: Increasing concentration led to decreased optical density, indicating bactericidal effects. Significantly lower optical density values were observed in decreasing order among the test samples (25, 50, and 100 μL) compared to control and antibiotic groups against Streptococcus, highlighting the potent antibacterial and antibiofilm properties of the greenly generated combination of titanium oxide NPs with herbs. This was also confirmed by moderate minimum inhibitory concentration at 100 μL.

Conclusion: The present study suggests that there is a bactericidal process at play, leading to a reduction in the overall bacterial count. It can be concluded that the ginger and rosemary-mediated titanium oxide NPs serve as potential antibacterial agents against S. aureus. This study can be used as a preliminary study, and further studies can be conducted to use this formulation in the field of medicine.

## Introduction

Indeed, the development of nanotechnology has led to significant advancements in various fields, including medicine, electronics, and materials science. Nanoparticles (NPs), defined as objects with at least one dimension ranging from 1 to 100 nanometers, have unique physicochemical properties compared to larger particles of the same composition. One of the significant advantages of NPs is their high surface-to-volume ratio [1]. Metal-based nanomaterials offer numerous advantages in the fields of biology and medicine. They can be engineered to have specific sizes, shapes, and surface properties, allowing the drugs or therapeutic agents to be delivered on a targeted level to specific cells or tissues. This targeted drug delivery system enhances the efficacy of treatments while minimizing side effects [2,3].

The green synthesis of NPs requires the selection of environmentally friendly solvents (such as water or ethanol), non-toxic reducing agents, and safe stabilizing substances. Traditional chemical routes for NP synthesis involve toxic and hazardous chemicals, posing environmental risks and high costs. In contrast, green synthesis offers a safe, biocompatible, and eco-friendly approach to producing NPs for various applications, including biomedicine [4]. Plant-derived NPs offer a distinct advantage over chemically synthesized NPs due to their reduced likelihood of inducing harmful side effects. These green NPs exhibit remarkable biological potential, making them valuable in various fields such as agriculture, food science, bioengineering, cosmetics, nanomedicine, and safeguarding human health [5]. 

Titanium oxide (TiO_2_) or titania can be found as a thin film or as a fine, white powder. TiO_2_, brookite, anatase, and rutile are the four naturally occurring crystal forms of TiO_2_. TiO_2_ NPs are renowned for their desirable qualities, which include mechanical, electrical, optical, rheological, thermal, physicochemical, and biological properties. With their antimicrobial properties, TiO2 NPs effectively combat oral pathogens associated with dental caries and periodontal diseases. They disrupt bacterial cell walls and membranes, leading to increased permeability and cell death [4]. The remarkable antimicrobial properties of TiO_2_ NPs render them exceptionally valuable in restorative materials, dental adhesives, sealants, cements, and various other dental applications. By incorporating TiO_2_ NPs, dental products can effectively prevent microbial colonization, thus significantly reducing the risk of infections [6]. 

In dentistry, the success of restorative materials heavily relies on their biological strength as well as their mechanical, physical, and chemical properties. TiO_2_ NPs have been widely incorporated into various endodontic restorative materials displaying remarkably effective antimicrobial activity. Within oral dentifrices, TiO_2_ NPs are harnessed in prophylactic measures against sensitivity and caries, typically at concentrations ranging from 1% to 10% by weight. The mean particle sizes of these NPs typically fall between 100 and 300 nm, which ensures their convenient solubility in enamel, dentin, and cementum. As a result, the incorporation of TiO_2_ NPs effectively serves as a preventive measure against the development of caries and tooth sensitivity. Given these advantageous properties, the utilization of these NPs is strongly recommended in the realm of current adhesive dentistry practices. By embracing such advanced nanotechnologies, dental care can be enhanced, and the prevalence of oral health issues can be significantly reduced, paving the way for more promising and effective oral healthcare strategies [7].

Throughout various cultures, the botanical treasure known as ginger (Zingiber officinale) has been esteemed for its medicinal virtues, with a history of utilization spanning centuries. This esteemed herb has garnered a reputation for its multifaceted potency, particularly in its role as an antimicrobial agent, among other remarkable biological activities. These exceptional attributes are attributed to the presence of bioactive compounds such as gingerol, paradol, shogaols, and zingerone, which collectively contribute to the vast array of therapeutic benefits found within this botanical gem. The diverse pharmacological potential of ginger continues to captivate researchers and practitioners alike, offering promising avenues for harnessing its powerful natural remedies in a plethora of health applications and holistic wellness practices. Notably, a study highlighted the antimicrobial potential of a 10% ethanolic ginger extract against pathogens [8]. Furthermore, ginger-based NPs have shown synergistic effects when combined with conventional antibiotics, enhancing their antibacterial efficacy and overcoming bacterial resistance. This suggests their potential application in combating drug-resistant bacterial strains [9]. Similarly, rosemary (Rosmarinus officinalis), an aromatic shrub belonging to the Lamiaceae family, has been traditionally used for its hepatoprotective and antiangiogenic properties and therapeutic effects on Alzheimer's disease. The antibacterial activity of rosemary is due to the presence of rosmarinic acid, epirosmanol, rosmaridiphenol, carnosic acid, carnosol, rosmanol, and isorosmanol. These compounds interact with the cell membrane, causing genetic changes and cellular alterations [10,11].

The primary aim of the present study is to formulate an antibacterial concoction by incorporating extracts from ginger and rosemary, along with titanium oxide NPs. The study seeks to explore the synergistic antibacterial potential of these natural extracts and NPs against Staphylococcus aureus. The objectives of the study are to extract bioactive compounds from ginger and rosemary, synthesize titanium oxide NPs, and combine them to create an antibacterial mixture. To characterize the concoction's chemical composition, NP dispersion, and antibacterial activity against S. aureus and to investigate the synergistic effects of minimum inhibitory concentration (MIC) and minimum bactericidal concentration (MBC), and antibacterial mechanisms contributing to understanding natural extract and NP applications. Through achieving these objectives, this study aims to provide insights into the feasibility of utilizing ginger and rosemary extracts in combination with titanium oxide NPs for developing effective antibacterial agents against S. aureus, potentially leading to novel strategies in combating bacterial infections in the oral cavity.

## Materials and methods

Study setting

The investigation was conducted within the Nanomedicine Laboratory, situated in the Department of Pharmacology at Saveetha Dental College and Hospitals, which is part of Saveetha Institute of Medical and Technical Sciences located in Chennai, Tamil Nadu, India. Prior to commencing the study, approval was obtained from the Scientific Review Board (SIMATS/Ph.D.Regn./A4/2022/406-06). The experiment was initiated in June 2023 and was conducted within a time period of two weeks from acquiring the raw products, titanium dioxide NPs, and performing antibacterial tests.

For the current study, the oral pathogen S. aureus was utilized to assess the antibacterial activity and was sourced from the Nanobiomedicine Laboratory's culture laboratory which is affiliated with Dental College and Hospitals, Saveetha Institute of Medical and Technical Sciences, Chennai, Tamil Nadu, India.

Preparation of the plant extract

The raw products for the preparation of plant extract were acquired from a local market. One gram of ginger (Zingiber officinale) and one gram of rosemary powder (Rosmarinus officinalis L.) were taken and mixed together with a quantity of 100 mL of distilled water. The solution was boiled at 600 ℃ for a period of 10-15 minutes. The solution was filtered into a flask using No.1 size Whatman filter paper. The final extract was cooled to room temperature and was stored in an airtight storage box.

Synthesis of TiO_2_ NPs

**Figure 1:**
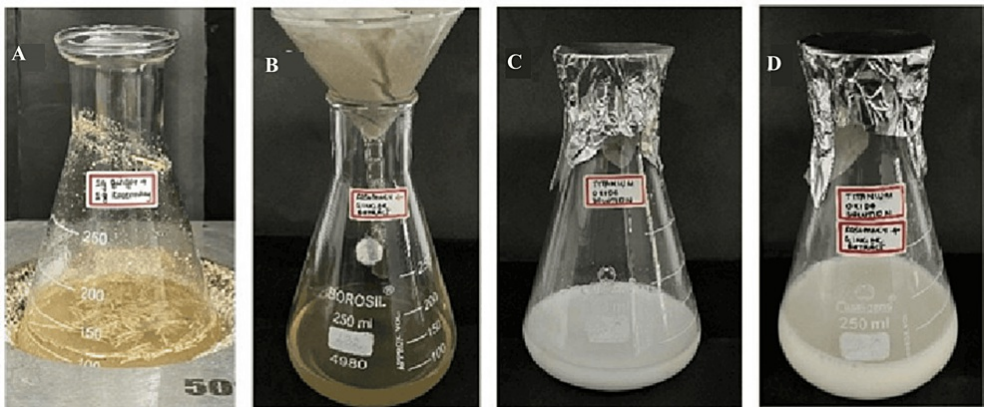
Pictorial representation of the process of formulation of TiO2 nanoparticle-mediated rosemary and ginger extract. (A) Mixture of ginger and rosemary powder; (B) rosemary and ginger plant extract; (C) titanium oxide solution; (D) synthesis of titanium oxide and plant extract preparation

For the synthesis of TiO_2_ NPs, TiO_2_ powder of 6.26 mM was taken; 0.5 grams of TiO_2_ powder was dissolved in 50 mL of distilled water to be made as a precursor. Fifty milliliters of the prepared rosemary and ginger extract was added to the prepared mixture. The solution was kept in a magnetic stirrer for 48 hours. The change in the color of the solution was observed, and readings were taken using a UV spectrometer. After the synthesis of NPs, it was centrifuged for 10 minutes. The pellets of TiO_2_ NPs enriched with ginger and rosemary were collected and used for further analysis (Figure 1). The stock solution will be diluted as a working solution of different concentrations (25, 50, and 100 μL) before antibacterial testing.

Antibacterial activity

**Figure 2:**
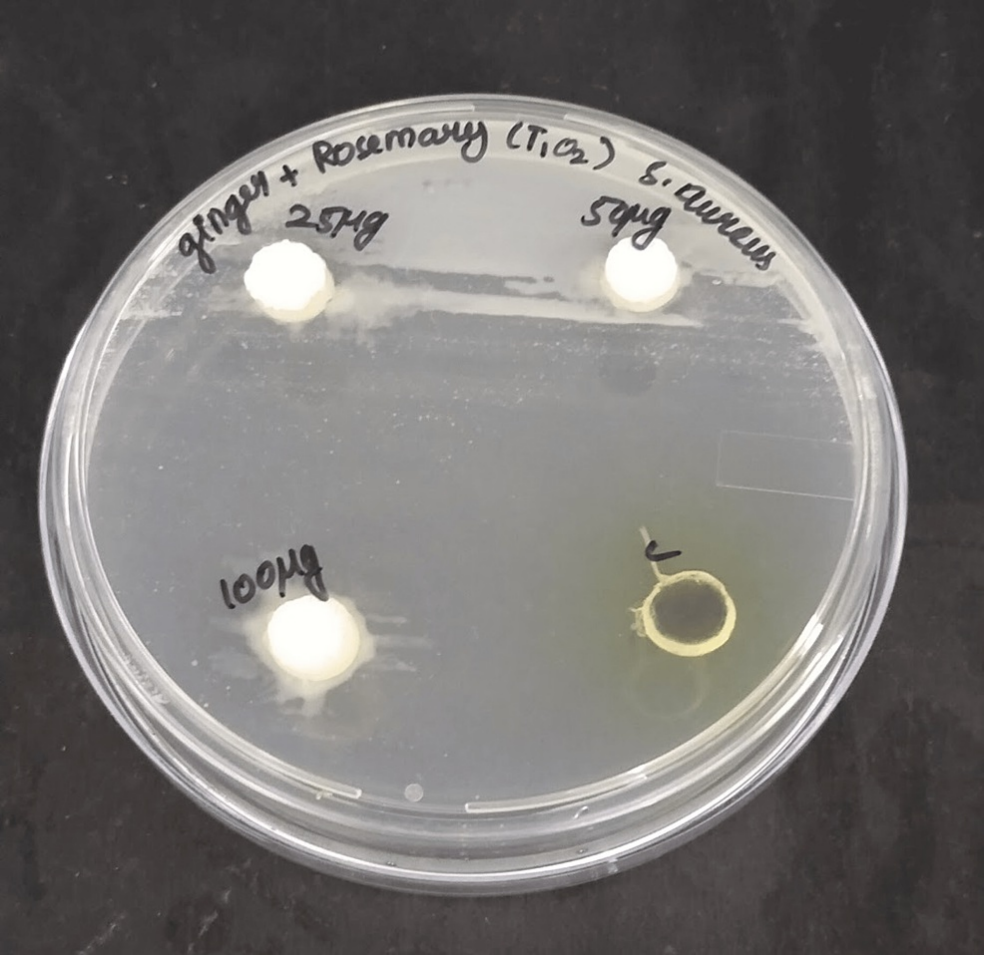
The zones of inhibition for Staphylococcus aureus at various TiO2-NPs clove-ginger concentrations

The antimicrobial activity of the TiO_2_ reinforced with ginger and rosemary extract was tested against S. aureus strains. The Muller-Hinton Agar was prepared and it was used to identify the zone of inhibition by the bacteria. After serially diluting the test solution (25, 50, and 100 μL) with 50% water, it was added to well culture plates that contained a four-hour culture of S. aureus at 370C. The sample grouping was a test group and a positive control group. The number of test samples used was 4 for each concentration i.e. 25, 50, and 100 μL, respectively. The positive control group antibiotic was Amoxyrite. The culture plate was incubated and the readings of the zone of inhibition were noted (Figure 2).

The collected data were entered in Microsoft Excel (Microsoft Corporation, Redmond, Washington) and then exported to IBM SPSS Statistics for Windows, Version 25 (Released 2017; IBM Corp., Armonk, New York, United States) for statistical analysis. The level of significance was <0.005 for one-way ANOVA.

## Results

**Figure 3:**
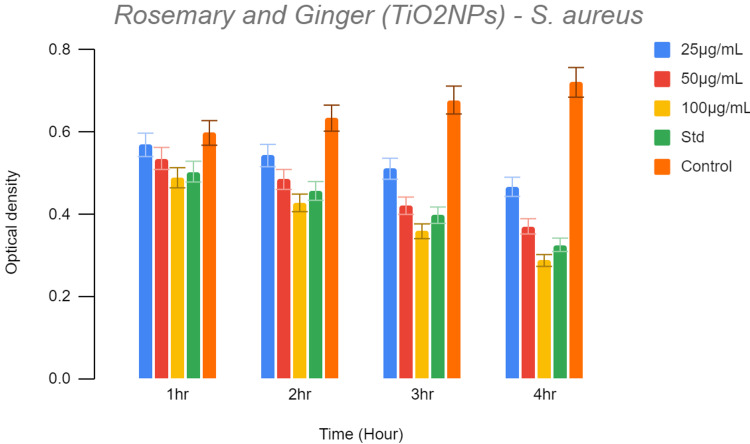
The graph depicts the antibacterial activity of our preparation The graph depicts the antibacterial activity of titanium oxide nanoparticle-infused rosemary and ginger solution against S. aureus at 1, 2, 3, and 4 hours of incubation

The phenomenon of optical density entails the measurement of light scattering produced by bacteria present within a culture plate. As the concentration of bacteria increases, the scattering of light also intensifies proportionally. As the graph (Figure 3) depicts, the first hour shows high optical density that is found to be on par with control; 25μL shows increased optical density and it gradually decreases on 100μL. As the time increases, the optical density decreases even with the lowest test solution.

The concentration with the least optical density indicates less bacterial growth and high bactericidal activity in comparison with the control (Table 1). 

**Table 1:** Recorded optical density reading of the mixture containing titanium oxide nanoparticle-infused rosemary and ginger against S. aureus at different concentrations (25, 50, 100 μL, standard, and control)

	1hr	2hr	3hr	4hr
25µg/mL	0.569	0.543	0.511	0.467
50µg/mL	0.536	0.485	0.421	0.371
100µg/mL	0.489	0.428	0.359	0.288
Std	0.504	0.457	0.398	0.326
Control	0.598	0.634	0.678	0.721

The experiment aimed to assess the impact of different concentrations of TiO_2 _NPs, specifically at 25 μL, 50 μL, and 100 μL dilutions, on the growth inhibition of S. aureus. The zones of inhibition, which indicate the effectiveness of the test solutions in restraining bacterial growth, were observed and compared. At 25 μL dilution of TiO_2_ NPs, the zone of inhibition observed for S. aureus was relatively less evident. This suggests that at this lower concentration, the TiO_2_ NPs may have a limited inhibitory effect on bacterial growth. Comparatively when the test solution was prepared with a 50 μL dilution of TiO_2_ NPs, a similar outcome was observed, with a less evident zone of inhibition for S. aureus implying that even at a slightly higher concentration, the inhibitory effect of the TiO_2_ NPs remains relatively modest. However, at 100 μL dilution of TiO_2 _NPs, a more promising result emerged. S. aureus displayed a moderately sized zone of inhibition, indicating that at this concentration, the TiO2-NPs exhibited a stronger inhibitory effect on bacterial growth compared to the lower dilutions. Lastly, the control experiment involving Amoxyrite showed a moderately sized zone of inhibition as well, serving as a reference point for comparison.

In summary, the experiment demonstrated that the effectiveness of TiO_2_-NPs in inhibiting S. aureus growth is concentration-dependent. While lower dilutions exhibited less evident inhibition, a 100 μL dilution of TiO_2_-NPs showed a moderately sized zone of inhibition, indicating potential antibacterial properties comparable to the control.

## Discussion

In recent times, there has been a growing fascination among researchers with diverse plant-derived compounds in the scientific community. Natural products are attracting significant attention due to their remarkable tolerance profiles compared to some synthetic alternatives, making them an essential source of potential drugs. One prominent aspect of these natural compounds is their ability to act as antimicrobial agents, providing valuable means to control various microorganisms.

These botanical wonders have a wide range of benefits, with their antimicrobial properties being particularly crucial in fields like pharmacology, medicine, and agriculture. Scholars are eager to understand the intricate mechanisms and therapeutic potentials of these bioactive substances, as they offer promising solutions to combat microbial challenges. Exploring nature's pharmacy opens up new possibilities for innovative and sustainable healthcare solutions. Hence, we have embraced the concept of green synthesis in the current study which enables us to capitalize on the advantages of NPs while concurrently mitigating environmental impacts and fostering sustainable practices for a greener future [5]. S. aureus despite being a human pathogen is also regarded as a commensal organism. Although it colonizes other anatomical areas, including the mouth, S. aureus is primarily isolated from the anterior nares while acting as a commensal [12].

Numerous scholarly works have examined the antimicrobial effectiveness of titanium dioxide NPs against a range of bacteria, encompassing both Gram-positive and Gram-negative bacteria. Some studies have reported promising results, demonstrating the ability of TiO2 NPs to inhibit bacterial growth and effectively kill bacteria. Titanium dioxide NPs have been widely studied for their antibacterial properties [13]. Haghi et al. conducted a study to assess the antimicrobial effect of TiO_2_ NPs on a pathogenic strain of Escherichia coli. Their research revealed that TiO2 NPs induce the formation of tiny pores in the bacterial cell walls, resulting in heightened permeability and ultimately leading to cell death. This mechanism contributes to the impressive antimicrobial efficacy of TiO_2_ NPs, making them a promising option for combating microbial threats in dental settings [6]. This correlates with the positive results of the current study using titanium oxide NPs as one of the major compositions of the concoction toward an effective antimicrobial action. The antibacterial efficacy of TiO_2_ NPs is primarily attributed to their photocatalytic properties. On exposure to ultraviolet (UV) light, TiO_2_ NPs can generate reactive oxygen species (ROS), like hydroxyl radicals consisting of strong oxidative capabilities. These ROS can cause damage to bacterial cell membranes and biomolecules, leading to cell death [14].

In addition to this, a study by Khashan et al. also represents the optical density of TiO_2_ NPs against various bacterial strains. As the concentrations of TiO_2_ NPs increased, the optical density exhibited a slight decrease across all the tested strains. According to Khashan et al., the optimal concentration of TiO_2_ NPs to prevent bacterial growth was discovered to be at 1000 µg mL^-1^ with an inhibition rate of 42, 25, 15, 30% 42%, 25.5%, 15.05%, and 30.5% respectively, whereas the inhibition rates of TiO_2_ NPs at 400 µg mL-1 were 21%, 3.5%, 5.3%, and 4.9% against E. coli, S. aureus, Pseudomonas aeruginosa, and P. vulgaris, respectively. According to these investigations, titanium oxide itself has bactericidal properties against several bacterial strains [15].

Another article of 2023 suggests that TiO_2_ NPs augmented with clove and ginger may be employed as an antibacterial agent against Lactobacillus species. This indicates that the TiO_2_ NPs serve as a potential candidate as an antibacterial agent against lactobacillus [16]. In the early days of NP synthesis, Senapati et al. conducted a study on gold and silver NPs and demonstrated their structural properties toward biomedical applications, which played an important role in considering TiO_2_ NPs to study their antimicrobial properties. The majority of the studies illustrating the antibacterial activity of the NPs were demonstrated by suspension of TiO_2_ into the solution and thereby studied bacterial inhibition and its activity [17]. In a study conducted by Rezaei et al., where they compared the antibacterial efficiency against E. coli between TiO_2_ NPs and cadmium oxide NPs, the TiO_2_ NPs had a better antibacterial activity than the cadmium oxide NP, and 0.1% of TiO2 NPs inhibited bacterial growth that was tested on E. coli. This evidence substantiates the superior benefits of titanium oxide NPs, making them a more favorable choice for exploring metallic oxide NPs in the creation of novel dental varnishes incorporating both NPs and herbal ingredients [18].

Conducting in vivo studies, involving animal models or human subjects, is essential for a more realistic understanding of how these NPs behave within biological systems. Understanding the precise interactions between the NPs and bacterial cells, as well as the specific cellular pathways affected, could provide deeper insights into potential applications.

Limitations

The study's scope is limited as it solely focuses on the antibacterial effectiveness of the synthesized NPs against S. aureus. While S. aureus is important, a comprehensive evaluation should encompass a broader spectrum of bacterial strains, including both Gram-positive and Gram-negative types. The study is conducted entirely in a controlled laboratory environment, which is valuable for initial insights. However, it lacks the complexity of interactions occurring within living organisms. The study also lacks a thorough exploration of the mechanisms driving the observed antibacterial activity.

## Conclusions

The investigation establishes the bactericidal efficacy of a combination comprising ginger and rosemary-infused titanium oxide NPs, resulting in a reduction of bacterial counts when evaluated across varying concentrations (25, 50, and 100 μL) in relation to S. aureus. The results have proved that as the bacterial concentration increased, there was a proportional intensification in light scattering indicating that the optical density was notably high and comparable to the control group which gradually when the concentration reached 100μL. This was supported by the values of zones of inhibition with moderate inhibition at the concentration 100μL in comparison with 25 and 50 μL concentrations of the herbal and nanoparticle mixture. This preliminary research lays the foundation for future medical applications, warranting subsequent investigations for practical use in the field of medicine.
